# Rev-erbα agonists suppresses TGFβ1-induced fibroblast-to-myofibroblast transition and pro-fibrotic phenotype in human lung fibroblasts

**DOI:** 10.1016/j.bbrc.2023.05.092

**Published:** 2023-05-24

**Authors:** Chandrashekhar Prasad, Kameron Hahn, Santosh Kumar Duraisamy, Matthias A. Salathe, Steven K. Huang, Thomas P. Burris, Isaac Kirubakaran Sundar

**Affiliations:** a Department of Internal Medicine, Division of Pulmonary Critical Care and Sleep Medicine, University of Kansas Medical Center, Kansas City, KS, USA; b Division of Biological Sciences, University of Missouri, Columbia, MO, USA; c Department of Internal Medicine, Division of Pulmonary and Critical Care Medicine, University of Michigan, Ann Arbor, MI, USA; d College of Pharmacy, University of Florida, Gainesville, FL, USA

**Keywords:** Pulmonary fibrosis, TGFβ, Collagen, Circadian clock, Rev-erbα agonist, SR9009, GSK4112, SR8278

## Abstract

Idiopathic pulmonary fibrosis (IPF) is an interstitial lung disease characterized by excessive scarring of the lungs that can lead to respiratory failure and death. Lungs of patients with IPF demonstrate excessive deposition of extracellular matrix (ECM) and an increased presence of pro-fibrotic mediators such as transforming growth factor-beta 1 (TGFβ1), which is a major driver of fibroblast-to-myofibroblast transition (FMT). Current literature supports that circadian clock dysfunction plays an essential role in the pathophysiology of various chronic inflammatory lung diseases such as asthma, chronic obstructive pulmonary disease, and IPF. The circadian clock transcription factor Rev-erbα is encoded by *Nr1d1* that regulates daily rhythms of gene expression linked to immunity, inflammation, and metabolism. However, investigations into the potential roles of Rev-erbα in TGFβ-induced FMT and ECM accumulation are limited. In this study, we utilized several novel small molecule Rev-erbα agonists (GSK41122, SR9009, and SR9011) and a Rev-erbα antagonist (SR8278) to determine the roles of Rev-erbα in regulating TGFβ1-induced FMT and pro-fibrotic phenotypes in human lung fibroblasts. WI-38 cells were either pre-treated/co-treated with or without Rev-erbα agonist/antagonist along with TGFβ1. After 48 h, the following parameters were evaluated: secretion of COL1A1 (Slot-Blot analysis) and IL-6 (ELISA) into condition media, expressions of α-smooth muscle actin (αSMA: immunostaining and confocal microscopy), and pro-fibrotic proteins (αSMA and COL1A1 by immunoblotting), as well as gene expression of pro-fibrotic targets (qRT-PCR: *Acta2, Fn1,* and *Col1a1*). Results revealed that Rev-erbα agonists inhibited TGFβ1-induced FMT (αSMA and COL1A1), and ECM production (reduced gene expression of *Acta2, Fn1,* and *Col1a1*), and decreased pro-inflammatory cytokine IL-6 release. The Rev-erbα antagonist promoted TGFβ1-induced pro-fibrotic phenotypes. These findings support the potential of novel circadian clock-based therapeutics, such as Rev-erbα agonist, for the treatment and management of fibrotic lung diseases and disorders.

## 1. Introduction

Idiopathic pulmonary fibrosis (IPF) is a progressive fibrotic lung disease, marked by extensive remodeling and scarring of the lungs that, in some cases leads to respiratory failure and death [1]. Fibroblast-to-myofibroblast transition (FMT) is a key process that promotes IPF pathobiology. Transforming growth factor-beta 1 (TGFβ1), a major pro-fibrogenic cytokine that drives FMT, is a central player in the pathogenesis of IPF [2,3]. Although myofibroblast activation is essential for normal wound healing, recurrent micro-injury of the lung alveolar epithelium can result in an exaggerated response that leads to increased ECM accumulation and decreased degradation/clearance that ultimately culminates in fibrosis [2–4]. Myofibroblasts are the key effector cells responsible for fibrogenesis via expression of α-smooth muscle actin (αSMA), extracellular matrix production including fibronectin (FN1) and collagens, and promoting secretion of pro-fibrotic cytokines (e.g., IL-6, IL-1β, etc.) [4–6].

Mounting evidence supports the role circadian clock proteins in the pathobiology of chronic lung diseases including IPF [7–10]. Circadian clocks are endogenous oscillators that control 24-h physiological and behavioral processes in mammals, regulated externally by Zeitgebers (time of feeding, exercise, sleep-wake cycle, etc.) and intracellularly by a network of core clock genes refer to as activators (circadian locomotor output cycles kaput [*CLOCK*] and brain and muscle ARNT-like 1 [*BMAL1*]), repressors (Periods [*PER1/2/3]* and Cryptochromes [*CRY1/2*]) and other clock genes (nuclear receptor subfamily 1 group D member 1/2 [*NR1D1/2* or *REV-ERBα/β*], RAR-related orphan receptors [*RORα/γ*], and nuclear factor interleukin 3 regulated [*NFIL3*]) [7]. RORs and REV-ERBs participate in an accessory or secondary loop of the circadian clock by competitively binding to the ROR response element (RORE) to either activate or repress the transcription of *Bmal1*. Previous studies have shown that synthetic ligands/Rev-erbα agonists (e.g., GSK4112, SR9009, SR9011, etc.) that activate Rev-erbα promote the suppression of Rev-erbα-dependent target genes, and Rev-erbα antagonist (SR8278) inhibits the Rev-erbα-mediated suppression of target genes [11,12]. Earlier reports have demonstrated Rev-erbα agonists as a novel therapeutic agent that can modulate cellular and physiological processes linked to inflammation and metabolism [11,13–16]. Clock mutant (*Clock*^*∆19*^) and *Rev-erbα* fibroblast-specific knockout (KO) mice demonstrate worse fibrosis after bleomycin-induced lung injury. Furthermore, treatment with *Rev-erbα* agonist GSK4112 or SR9009 reduced the fibrotic responses in human lung fibroblasts, *ex-vivo* lung tissue explant cultures, and prophylactic model of bleomycin-induced lung fibrosis [8–10].

This study comprehensively evaluated the potential roles of different *Rev-erbα* agonists and antagonists in modulating TGFβ1-induced FMT in human lung fibroblasts. We have utilized Rev-erbα agonists GSK4112, SR9009, SR9011, and antagonist SR8278 as a novel clock modulator that can attenuate TGFβ1-induced pro-fibrotic phenotypes in human lung fibroblasts (WI-38 cells).

## 2. Materials and methods

### 2.1. Materials

Materials for protein separation using SDS-polyacrylamide gel such as 4X Laemmli buffer (Cat#:1610747), 40% acrylamide/Bis solution 37.5:1 (Cat#:1610148), *Trans*-blot turbo protein transfer system (Cat#:1704271) were purchased from Bio-Rad, USA. Sodium dodecyl sulfate (Cat#: 28365) from Thermo Scientific, USA. Cell culture medium DMEM: F12 (Cat#: 16-405-CV) was obtained from Corning Life Sciences, USA, fetal bovine serum (Cat#: SH30910.03), and nitrocellulose membrane (0.2 μm, Amersham^™^) from Cytiva, and antibiotic-antimycotic solution (Cat#: 15240-062, Streptomycin sulfate, Penicillin G sodium, and Amphotericin), and 0.05% trypsin-EDTA (Cat#: 25300–054) solution from Gibco purchased through Thermo Scientific, USA. Primary antibodies COL1A1 (E8F4L, Rabbit mAb; Cell Signaling Technology, USA), and COL1A1 (Cat#:SC-293182, Mouse mAb; Santa Cruz Biotechnology, USA), HRP-conjugated secondary antibodies (Goat anti-Rabbit IgG H + L, Cat#: 65–6120 and Goat anti-Mouse IgG, H + L, Cat#: 31431) and fluorescently labeled secondary antibodies (Alexa fluor 555; Cat#: A-21422, Goat anti-Mouse, IgG H + L), ProLong^™^ Gold-antifade reagent with DAPI (Cat#: P36935), Alexa Fluor^™^ 488 phalloidin (Cat#: A12379) were purchased from Invitrogen, USA. Pierce BCA Assay Kit (Cat #: 23225) and western blotting chemiluminescent substrate SuperSignal^™^ West Pico (Cat#:34577) and West Femto (Cat#: 34096) were procured from Thermo Scientific. The Rev-erbα agonists GSK4112 (Cat#: 3663), SR9009 (Cat#:5855), SR9011 (Cat#: 11930), and antagonist SR8278 (Cat#: 4463) were obtained from TOCRIS Bioscience or Cayman Chemicals, USA. AO/PI staining solution (Cat#: CS2-0106) was obtained from Nexcelom Bioscience, USA. Slot-Blot manifold (Cat#: PR648) apparatus was purchased from Hoefer Inc. USA. Primary antibody αSMA (Cat#: A2547, Mouse mAb), protease and phosphatase inhibitor cocktail (Cat #: PPC1010) were obtained from Sigma, USA. ELISA assay kit for human IL-6 (Cat#: DY206) was obtained from R&D Systems, USA.

### 2.2. Cell culture

WI-38 human embryonic lung fibroblasts were obtained from (ATCC, USA), Normal (healthy: NHLF) and diseased (DHLF-IPF) were purchased from Lonza, USA. Cells were maintained in an incubator with a continuous supply of 5% CO_2_ (for WI-38 cells) or 5% CO_2_ and 3% O_2_ (for NHLF and DHLF) in humidified conditions (75–80% relative humidity). Cells were grown in DMEM:F12 (50:50 mix) culture medium supplemented with 10% fetal bovine serum (FBS) and 1% antibiotic-antimycotic. For serum starvation complete medium containing 1% FBS and 1% antibiotic-antimycotic were used. WI-38 cells were sub-cultured in a T75 flask and briefly treated with trypsin/scarped using a cell-scrapper and centrifuged (2000×*g* for 5 min). Cell viability was determined using AO/PI staining solution before every passage and seeding.

### 2.3. TGFβ1 treatment in human lung fibroblasts

WI-38 cells were seeded (5 × 10^5^ cells/well) in 6 well plates and maintained until they reached ~80% confluency. Then, cells were serum starved overnight (~16 h) followed by pre-treatment with the Rev-erbα agonists GSK4112 (20 μM for 1 h or 10 μM for 4 h) or SR9009 or SR9011 (10 μM for 4 h) or the Rev-erbα antagonist SR8278 (10 μM for 4 h). After pre-treatment, cells were washed with sterile phosphate buffered saline (1x PBS, pH 7.4) and then treated with or without TGFβ1 (5 or 10 ng/ml) for 48 h in complete medium (DMEM:F12). Cells were scrapped, and centrifuged (12,000×*g* for 5 min), and the pellets were stored at −80 °C till further analysis. The conditioned medium was collected and centrifuged (12,000×*g* for 5 min) to remove cell debris and stored at −80 °C for further analysis.

### 2.4. Slot-blot analysis

The frozen conditioned medium was thawed on ice and then assayed using a Slot-Blot apparatus to determine the relative release of secretory collagen (COL1A1). In brief, the Slot-Blot manifold was assembled with the nitrocellulose membrane (pore size: 0.2 μm) and attached to the vacuum. An equal volume (~200 μl) of PBS was loaded into each slot and a vacuum was applied to allow the sterile PBS to pass through. The vacuum was turned off and we loaded an equal volume of condition medium (~60 μl) into each slot. The vacuum was turned on until all the sample passes through the membrane. Once again, the vacuum was turned off, we added 60 μl of the PBS and allowed it to pass through the membrane with the vacuum turned on for 5 min. Finally, the Slot-Blot assembly was removed to take the transferred membrane. The membrane was blocked (5% BSA in TBST) for 1 h followed by the addition of primary antibody (COL1A1, 1:10,000) in a blocking buffer (5% skimmed milk) for 2 h. Washed with TBST gently (Twice, for 5 min each) with regular agitation and added secondary antibody (1:20,000; HRP-conjugated, Goat IgG anti-mouse) for 1 h followed by washing with TBST (for 4 times, 10 min each). The membrane was then developed using a chemiluminescent substrate and imaged by the LI-COR (Odyssey Fc) imaging system. The intensity of the protein band was quantified by densitometry analysis using Image J software (NIH).

### 2.5. Immunostaining and confocal microscopy

We used 4 well chamber slides for cell treatment followed by immunostaining. After 48 h post-treatment, cells were washed with ice-cold 1X PBS and fixed using 4% formalin for 10 min at room temperature (RT). Fixed cells were permeabilized using 0.2% Triton X-100 solution in 1X PBS for 10 min at RT. Washed with 1X PBS and blocked using 5% BSA in PBST (0.05% Tween 20 in 1X PBS) for 1 h at RT followed by incubation with primary antibody (αSMA; 1:200) in blocking buffer overnight at 4 °C. Next day, cells were washed with PBST wash buffer five times followed by addition of secondary antibody (1:800, Alexa Fluor 555 labeled IgG Goat anti-mouse, H + L, Invitrogen) for 1 h at RT. Cells were washed with PBST wash buffer five times to remove the unbound secondary antibody. As needed, cells were washed twice with 1X PBS and stained for F-actin using Alexa Fluor^™^ 488 phalloidin (400X, 66 μM, stock in DMSO diluted to 1X PBS containing in 1% BSA) for 20 min at RT and washed thrice with 1X PBS. Finally, cells were mounted using ProLong^™^ Gold-antifade reagent with DAPI and imaged at 60× (with immersion oil) objective using a confocal microscope (Nikon, USA). Image J software (NIH) was used to determine the pixel intensity of the image and analyzed the treatment effect quantitatively.

### 2.6. Protein isolation and western blotting analysis

Total protein was isolated from the frozen cell pellet in RIPA buffer with protease and phosphatase inhibitor cocktail with 3–4 times continuous freeze-thaw cycles and intermittent vortexing followed by centrifugation at 12,000×*g* for 25 min. The concentration of total cell protein was determined by Pierce BCA Assay Kit. An equal amount of protein (30 μg/lane) was loaded into the well after mixing with 4x Laemmli sample buffer (1:3) and then separated through 7.5% SDS-polyacrylamide electrophoresis (SDS-PAGE) at 80 mV for 2 h. The gels with protein samples were transferred onto a polyvinylidene difluoride membrane (0.45 μm, Immobilon^®^ transfer membrane, PVDF, Merck) for 40 min using a semi-dry TransBlot^®^ Turbo^™^ transfer system (Bio-Rad, USA). The membranes were blocked with 5% BSA in TBST (Tris buffer saline with 0.1% Tween 20) at RT for 2 h, followed by primary antibody incubation (1:1,000 in 5% BSA, overnight at 4 °C). Membranes were washed with TBST, for 10 min each (4 times), and probed with a HRP-conjugated secondary antibody (1:10,000 in 5% BSA) for 2 h at RT. Finally, membranes were developed using a chemiluminescent substrate and imaged by the LI-COR imaging system (Odyssey Fc). Fold change was determined after the normalization of the target protein with loading control GAPDH. The intensity of the protein band was quantified by densitometry analysis using Image J Software (NIH).

### 2.7. Total RNA isolation and cDNA preparation

Cell pellets were thawed on ice and lysed with 700 μl of QIAzol lysis reagent using a vortex and total RNA was extracted using the miRNeasy kit (Qiagen, Valencia, CA) according to the manufacturer’s protocol. Additionally, DNase treatment was performed using DNase I (Cat#: 79254, RNase-free DNase Set, Qiagen) for each sample as per the manufacturer’s protocol. Following RNA extraction, the concentration and purity of RNA were determined by a Nanodrop spectrophotometer (Thermo Scientific, USA). Complementary DNA (cDNA) synthesis was performed using High-Capacity cDNA Reverse Transcription Kit (Thermo Scientific, USA). Briefly, 650 ng of purified RNA was mixed with 4 μl of 10x RT buffer, 1.6 μl of 25x dNTPs mix, 2 μl of MultiScribe Reverse Transcriptase, 4 μl of 10x RT random primers, and a variable amount of DNase/RNase free water to make the final volume to 40 μl. Then the reaction mixture was incubated in a thermal cycler at 25 °C for 10 min, 37 °C for 120 min, 85 °C for 5 min, and held at 4 °C. Following cDNA synthesis 60 μl of DNase/RNase-free water was added to make the final volume of 100 μl.

### 2.8. Quantitative real-time polymerase chain reaction (qRT-PCR) analysis

All the qRT-PCR reactions were performed using PowerTrack^™^ SYBR^™^ Green Master Mix (Thermo Fisher Scientific) and gene-specific primers of pro-fibrotic genes (*Col1a1*, *Fn-1,* and *Acta-2)* along with reference gene (*Gapdh)* using the CFX Opus 96 Real-time PCR System (Bio-Rad). Briefly, 2 μl of cDNA, 8 μl of master mix containing 5 μl SYBR green, 0.25 μl of 40x yellow sample buffer, 1 μl of primer (forward and reverse), and 1.75 μl of DNase/RNase-free water were mixed to make a final volume of 10 μl. The reaction mixture was amplified using CFX Opus 96 Real-time PCR System (Bio-Rad) to determine the expression level of target genes. The quantification cycle (Cq) value was used to calculate the fold change in target gene expression by the 2^−∆∆Ct^ method using *Gapdh* as stable housekeeping genes. qRT-PCR primer sequences are included in the Supplementary Table S1.

### 2.9. Enzyme linked immunosorbent assay (ELISA)

The level of pro-inflammatory cytokines (IL-6) in the conditioned medium/cell supernatant following Rev-erbα agonist/anatagonist treatment was measured using ELISA assay kit (Human IL-6 DuoSet ELISA, R&D Systems) according to manufacturer’s instructions as previously reported [17].

### 2.10. Statistical analysis

Statistical analysis for significance was calculated between more than two groups using one-way ANOVA using Tukey’s multiple-comparison test with the GraphPad Prism 9 (La Jolla, CA). *P* < 0.05 is considered statistically significant.

## 3. Results

### 3.1. TGFβ1-mediated secretion of COL1A1 is attenuated by pre-treatment with Rev-erbα agonists

**Fig. 1.**
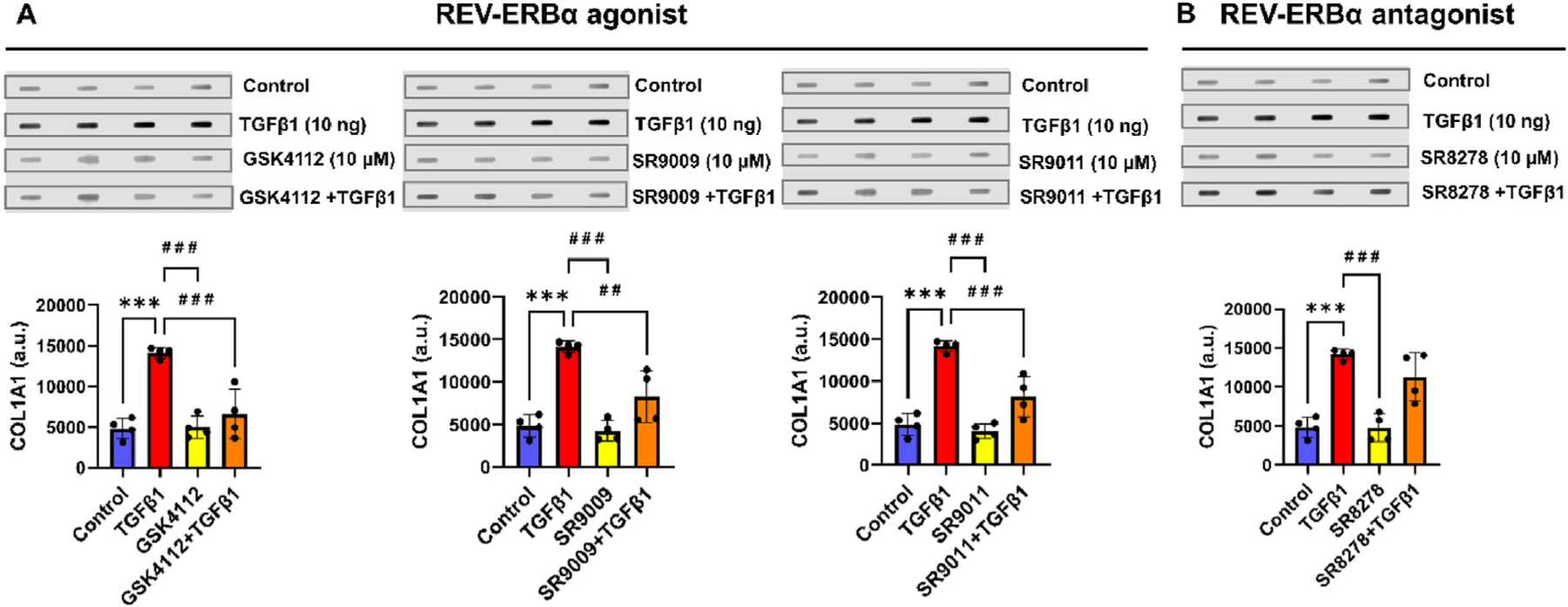
Rev-erbα agonist treatment inhibits TGFβ1-induced secretion of COL1A1 in WI-38 cells. (A–B) Human lung fibroblasts (WI-38 cells) were grown in 6 well plates serum-starved overnight and incubated with and without Rev-erbα agonist (GSK4112/SR9009/SR9011; 10 μM) or antagonist (SR8278; 10 μM) pre-treatment (for 4 h) before TGFβ1 (10 ng/ml) stimulation for 48 h. Conditioned medium was collected 48 h post-treatment and measured secretory COL1A1 using slot-blot analysis. Representative slot blot images were provided (each lane represents independent replicate samples). Control and TGFβ1 groups from the same experiment were used for all the different comparisons and densitometry of the bands was performed using Image J. Quantification of secretory COL1A1 is shown as arbitrary units (a.u.) in the bar graphs. Data are shown as mean ± SEM (n = 4/group); One-way ANOVA followed by Tukey’s multiple comparison test. *** *P* < 0.001, significant compared to the control group; ^# #^ *P* < 0.01, ^# # #^ *P* < 0.001 significant compared to TGFβ1 group.

Secretion of COL1A1 from WI-38 cells was determined by Slot-Blot analysis of the conditioned medium from control, TGFβ1 (10 ng/ml), Rev-erbα agonist (10 μM, GSK4112/SR9009/SR9011) or antagonist SR8278 (10 μM) alone, and Rev-erbα agonists or antagonist (pre-treatment for 4 h) followed by treatment with TGFβ1 for 48 h in WI-38 cells. Untreated control showed lower levels of baseline COL1A1 secretion and TGFβ1 treatment significantly increased COL1A1 secretion (Fig. 1 and Fig. S1). COL1A1 secretion was unchanged after treatments with Rev-erbα agonists (GSK4112, SR9009, SR9011) or antagonist (SR8278) alone. Pre-treatment for 4 h with Rev-erbα agonists followed by TGFβ1 treatment (GSK4112+TGFβ1, SR9009+TGFβ1, SR9011+TGFβ1) significantly reduced COL1A1 secretion compared to the TGFβ1-only treatment group. In contrast, pre-treatment with Rev-erbα antagonist followed by TGFβ1 treatment (SR8278+TGFβ1) showed increased COL1A1 secretion similar to what was observed in the TGFβ1-only treatment group (Fig. 1 and Fig. S1). Overall, these findings demonstrate that pre-treatment with Rev-erbα agonists suppressed secretion of COL1A1.

### 3.2. TGFβ1-mediated increase in αSMA was inhibited by Rev-erbα agonists

**Fig. 2.**
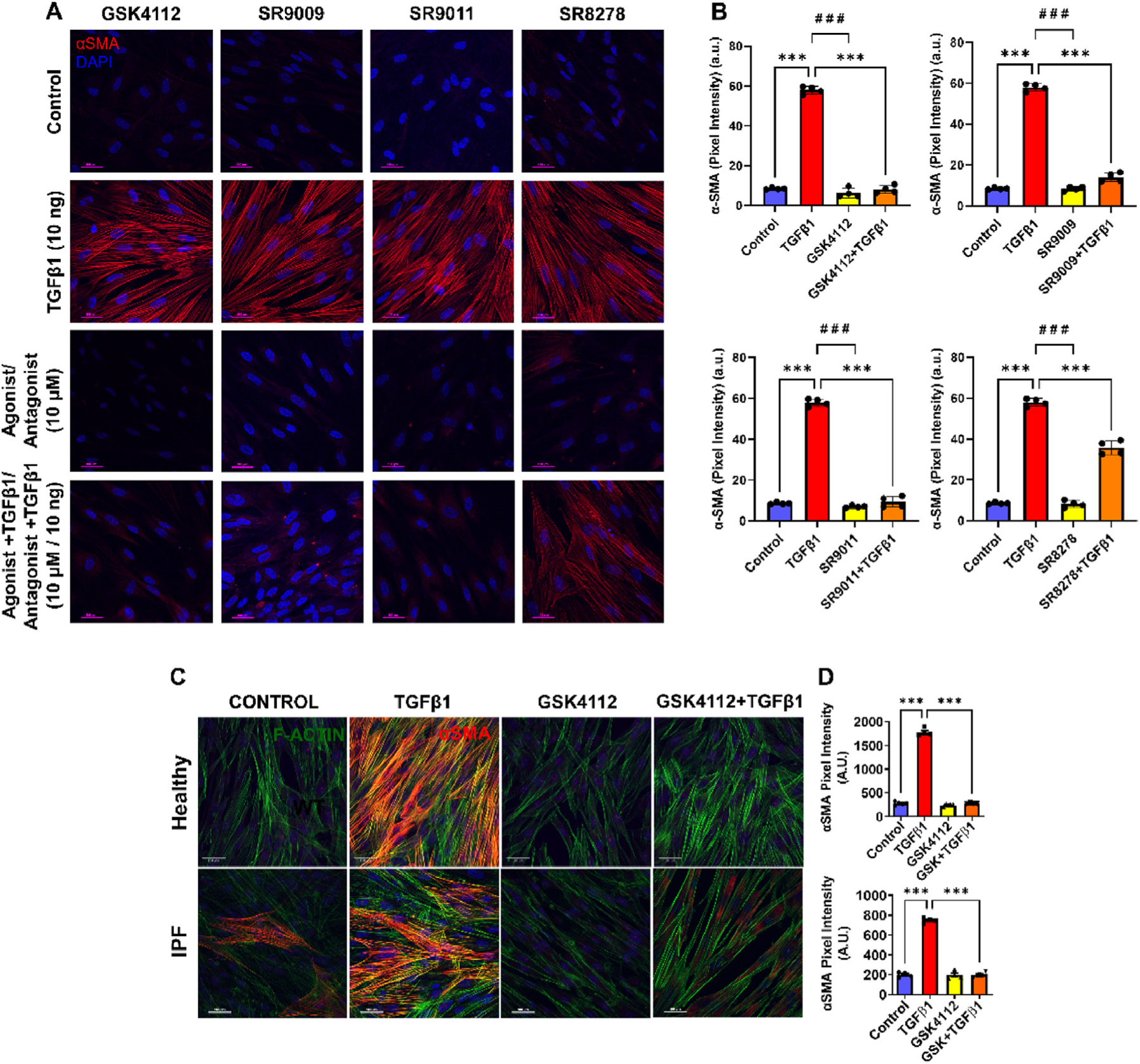
Rev-erbα agonist treatment inhibits TGFβ1-induced αSMA protein expression in human lung fibroblasts. Human lung fibroblasts (WI-38 cells) and primary human lung fibroblasts (healthy and IPF) were grown in 4 well chambered slides, serum-starved overnight, and incubated with and without Rev-erbα agonist (GSK4112/SR9009/SR9011; 10 or 20 μM) or antagonist (SR8278; 10 μM) pre-treatment (for 4 h) before TGFβ1 (10 ng/ml) stimulation for 48 h. (A) Representative images of WI-38 cells immunostaining showing αSMA (α smooth muscle actin) staining (red) and DAPI (blue) after TGFβ1 stimulation 48 h post-treatment using confocal microscopy (60x mag; scale bar: 100 μm). (B) Quantification of αSMA staining is shown as pixel intensity in the bar graphs. (C) Representative images of primary human lung fibroblasts from healthy and IPF immunostaining showing αSMA (red), F-actin/phalloidin (green), and DAPI (blue) after TGFβ1 stimulation 48 h post-treatment using confocal microscopy (60x mag; scale bar: 100 μm). (D) Quantification of αSMA staining is shown as pixel intensity in the bar graphs. Data are shown as mean ± SEM (n = 4/group); One-way ANOVA followed by Tukey’s multiple comparison test. *** *P* < 0.001, significant compared to the control group; ^# # #^ *P* < 0.001 significant compared to TGFβ1 group. (For interpretation of the references to colour in this figure legend, the reader is referred to the Web version of this article.)

TGFβ1-induced αSMA expression at 48 h post-treatment in WI-38 cells was confirmed by immunostaining and confocal microscopy. TGFβ1 treatment significantly increased αSMA abundance as evidenced by intracellular stress fibers stained in red (Fig. 2A–B). Treatment with each Rev-erbα agonist alone showed reduced αSMA expression within the cells, similar to the untreated control group. Pre-treatment with a Rev-erbα agonist followed by TGFβ1 treatment (GSK4112+TGFβ1 or SR9009+TGFβ1 or SR9011+TGFβ1) significantly reduced αSMA expression compared to TGFβ1-only treatment group. Rev-erbα antagonist (SR8278) alone also showed reduced αSMA expression compared to the control group; however, pre-treatment followed by TGFβ1 treatment (SR8278+TGFβ1) significantly augmented αSMA expression comparable to TGFβ1 alone treatment group (Fig. 2A–B). When primary human lung fibroblasts (HFL) from healthy (normal) and IPF (diseased) patients were treated with TGFβ1, intracellular αSMA expression (stress fibers) increased within the cells. Pre-treatment with Rev-erbα agonist GSK4112 (1 h) or GSK4112+TGFβ1 (1 h GSK4112 followed by TGFβ1 stimulation) significantly reduced TGFβ1-induced αSMA stress fibers in normal and IPF fibroblasts (Fig. 2C–D). Overall, these findings demonstrate that pre-treatment with Rev-erbα agonists significantly reduced intracellular αSMA abundance in WI-38 cells.

### 3.3. TGFβ1-mediated increase in COL1A1 and αSMA protein levels were attenuated by Rev-erbα agonists

**Fig. 3.**
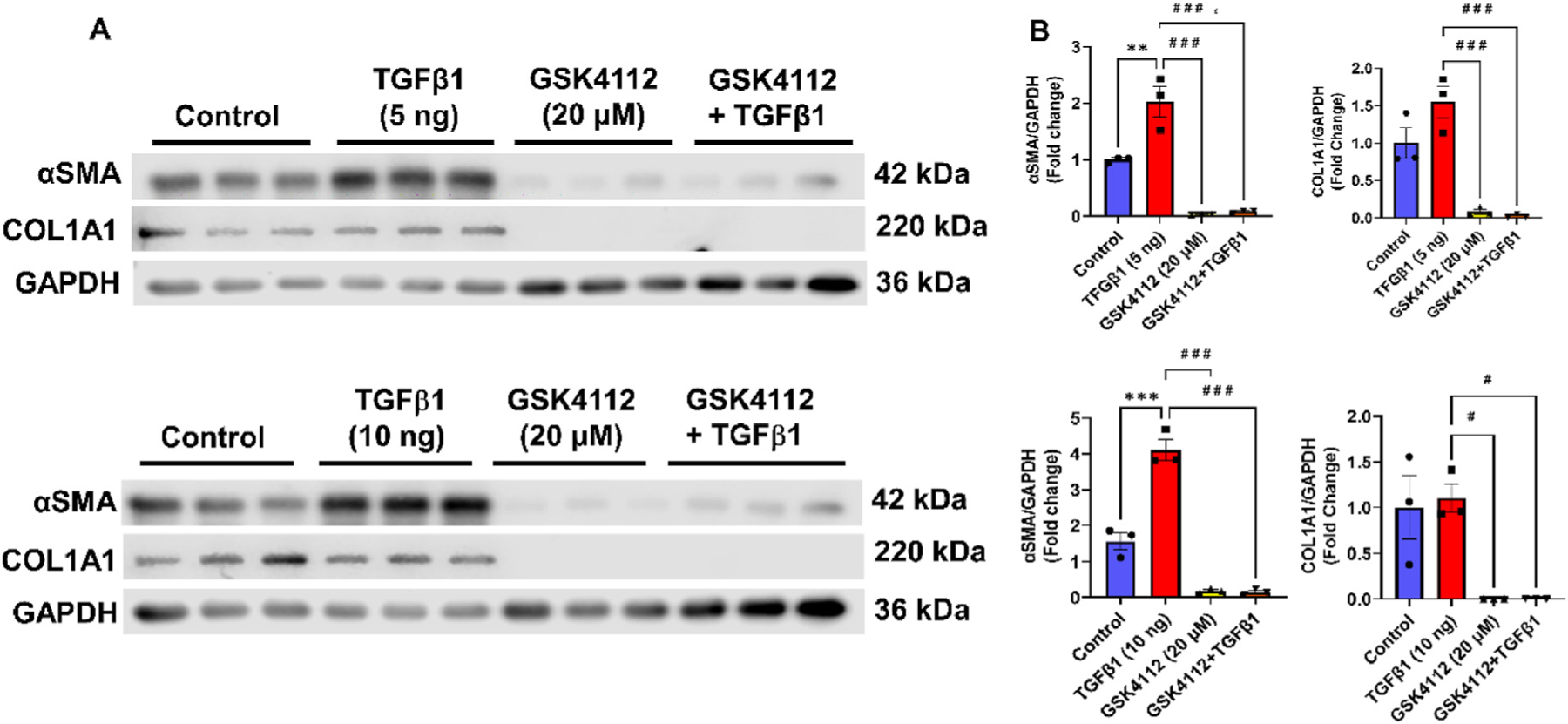
REV-Erbα agonist (GSK4112) inhibits TGFβ1-induced pro-fibrotic markers in WI-38 cells. Human lung fibroblasts (WI-38 cells) were serum-starved overnight and incubated with and without Rev-erbα agonist (GSK4112; 20 μM) pre-treatment (for 1 h) before TGFβ1 (5 ng/ml or 10 ng/ml) stimulation for 48 h. Whole-cell lysates were prepared to measure protein abundance of αSMA and COL1A1 by immunoblot analysis 48 h post TGFβ1 stimulation. GAPDH was used as housekeeping control. Representative WB images were provided, and densitometry of the bands was performed using Image J. Quantification of αSMA and COL1A1 protein levels is shown as fold change relative to the control group in the bar graphs. Data are shown as mean ± SEM; n ¼ 3/group. ** *P* < 0.01, *** *P* < 0.001, significant compared to control group; ^#^ *P* < 0.05, ^# # #^ *P* < 0.001, significant compared to TGFβ1 group.

Protein abundance of pro-fibrotic markers (αSMA and COL1A1) were analyzed using Western blotting of whole cell lysates 48 h post-treatment in WI-38 cells. TGFβ1, at both 5 and 10 ng/ml concentrations, significantly increased the protein levels of αSMA. Treatment with Rev-erbα agonist alone (GSK4112) and pre-treatment with GSK4112 for 1 h followed by TGFβ1 significantly reduced the protein level of αSMA compared to control and to TGFβ1 alone treatment groups (Fig. 3 and Figs. S2–S4). In contrast with the effect on αSMA, TGFβ1-only treatment did not increase the protein abundance of COL1A1 compared to control. However, pre-treatment with GSK4112 and GSK4112+TGFβ1 significantly reduced the protein levels of COL1A1 compared to TGFβ1 alone treatment group (Fig. 3 and Figs. S2–S4). Overall, we observed reduced αSMA and COL1A1 in both Rev-erbα agonist GSK4112 alone and GSK4112+TGFβ1 treatment groups as confirmed by Western blot analysis. These findings suggest a potential role of Rev-erbα agonist in blocking TGFβ1-induced expression of pro-fibrotic markers such as COL1A1 and αSMA in human lung fibroblasts.

### 3.4. TGFβ1-mediated activation of pro-fibrotic genes attenuated by Rev-erbα agonist treatment in human lung fibroblasts

**Fig. 4.**
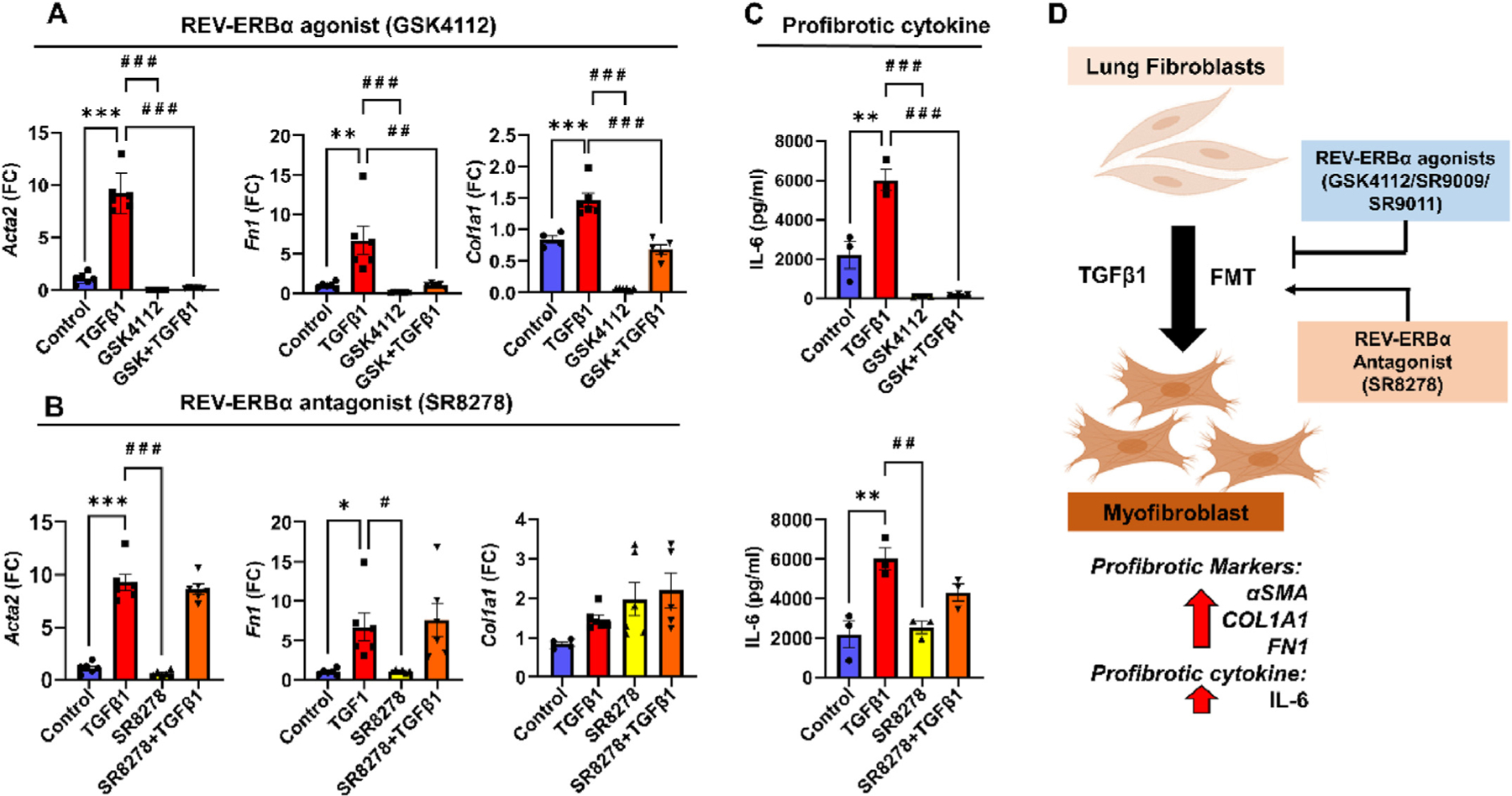
REV-Erbα agonist (GSK4112) inhibits TGFβ1-induced pro-fibrotic genes and cytokine in WI-38 cells. (A–B) Gene expression of *Acta2* (α smooth muscle actin)*, Fn1* (Fibronectin), and *Col1a1* (Collagen type I alpha 1 chain) were determined in REV-Erbα agonist/antagonist (GSK4112 20 μM; 1 h pre-treatment or SR8278 10 μM; 4 h pre-treatment) followed by addition of with or without TGFβ1 treatment for 48 h. GAPDH was used as the housekeeping gene and data were analyzed using the 2^−∆∆Ct^ method. (C) Pro-fibrotic cytokine IL-6 release in conditioned media was measured by ELISA after 48 h post TGFβ1 stimulation with and without GSK4112 or SR8278. (D) Schematic summary showing pre-treatment with Rev-erbα agonist inhibits TGFβ1-induced fibroblast-to-myofibroblast transition and pro-fibrotic phenotypes in human lung fibroblasts. Data are shown as mean ± SEM (n = 3e6/group); * *P* < 0.05, ** *P* < 0.01, ***P < 0.001, significant compared to control group; ^#^ *P* < 0.05, ^# #^ *P* < 0.01, ^# # #^ *P* < 0.001, significant compared to TGFβ1 group.

Transcript levels of pro-fibrotic genes (*Acta2, Col1a1,* and *Fn-1*) were analyzed using qRT-PCR 48 h post-treatment in WI-38 cells. TGFβ1 treatment augmented the transcript levels of pro-fibrotic genes *Acta2, Col1a1,* and *Fn-1* (Fig. 4A). Treatment with GSK4112 alone showed transcript levels of *Acta2* and *Fn-1* similar to the control group. Pre-treatment with the Rev-erbα agonist followed by TGFβ1 treatment (GSK4112+TGFβ1) significantly diminished transcript levels of all 3 pro-fibrotic genes *Acta2, Col1a1,* and *Fn-1* compared to TGFβ1 alone treatment group. Pre-treatment with Rev-erbα antagonist (SR8278) alone showed transcript levels of *Acta2* and *Fn-1* similar to control group, but *Col1a1* showed an increased trend. In contrast, pre-treatment with Rev-erbα antagonist followed by TGFβ1 treatment (SR8278+TGFβ1) augmented transcript levels of *Acta2, Col1a1,* and *Fn-1* comparable to TGFβ1 group (Fig. 4B). Overall, these findings provide additional evidence to support that TGFβ1-mediated activation of pro-fibrotic genes (*Acta2, Col1a1,* and *Fn-1*) were attenuated by pre-treatment/co-treatment with Rev-erbα agonist (GSK4112) but not Rev-erbα antagonist (SR8278) in human lung fibroblasts.

### 3.5. TGFβ1-mediated activation of pro-fibrotic cytokine IL-6 attenuated by Rev-erbα agonist treatment in human lung fibroblasts

TGFβ1-induced pro-inflammatory cytokine IL-6 release in conditioned media was measured 48 h post-treatment in WI-38 cells by ELISA. TGFβ1 treatment significantly increased IL-6 release compared to the control group. GSK4112 alone treatment reduced IL-6 release compared to the TGFβ1 treatment group. Pre-treatment with Rev-erbα agonist followed by TGFβ1 treatment (GSK4112+TGFβ1) significantly reduced IL-6 release compared to TGFβ1 alone group. Pre-treatment with Rev-erbα antagonist (SR8278) alone also reduced IL-6 release compared to the TGFβ1 treatment group (Fig. 4C). Furthermore, SR8278+TGFβ1 treatment showed a slight reduction in IL-6 release compared to TGFβ1 group that was not statistically significant. Overall, these findings suggest that GSK4112 treatment can potentially reduce TGFβ1-induced fibroblast-specific pro-inflammatory cytokines (e.g., IL-6) but not SR8278 treatment in human lung fibroblasts (Fig. 4D). Together these observations support that novel circadian clock-based therapeutics using Rev-erbα agonists can regulate pro-fibrotic pathways in human lung fibroblast with an implication for the treatment of pulmonary fibrosis.

## 4. Discussion

There is a strong connection between circadian clock dysregulation and the pathophysiology of chronic inflammatory diseases [13]. Aging is one of the major risk factors associated with pulmonary fibrosis (PF) [18,19]. Recent studies showed the molecular underpinnings between circadian clock dysregulation and the development of PF [8,9]. The nuclear receptor (NR) Rev-erbα is encoded by *Nr1d1* and regulates daily rhythms of gene expression linked to immunity, inflammation, and metabolism [11,15,17,20]. REV-Erbα and RORa are part of the accessory/secondary loop that plays an important role to control the first (core) loop. CLOCK:-BMAL1 heterodimer complex activates transcription of *Cry1/2*, *Per1/2/3*, *Rev-erbα/β,* and *Rorα/γ,* which results in daily fluctuation of core clock targets. Thus, activation of NRs (REV-Erbα and RORa) play an essential role in *Bmal1* transcription that regulates circadian rhythm in peripheral tissues [7]. This study provides additional evidence demonstrating that the circadian clock gene *Rev-erbα* regulates TGFβ1-induced FMT and ECM signaling in WI-38 cells. Novel clock modulators such as Rev-erbα agonists GSK4112, SR9009, and SR9011 can inhibit TGFβ1-mediated fibrotic response, whereas Rev-erbα antagonist (SR8278) further induces pro-fibrotic and pro-inflammatory phenotype in WI-38 cells.

TGFβ is a multifunctional fibrotic cytokine that is well-known to mediate FMT via activating TGFβ signaling pathways leading to excessive ECM accumulation followed by the progression of PF [2,3,5]. Currently, there are two FDA-approved anti-fibrotic drugs to treat IPF: Nintedanib, a tyrosine-kinase inhibitor, and Pirfenidone, an inhibitor of pro-fibrotic growth factors and collagens), both of which target TGFβ signaling. These drugs have been shown to reduce lung function decline; however, they do not halt the progression of IPF during end-stage disease [21,22]. Novel therapies to treat pulmonary fibrosis and other fibrotic diseases are an unmet clinical need that has to be addressed. Emerging studies using preclinical models, *in vitro* and *in vivo,* demonstrated the potential of circadian clock-based therapeutics such as Rev-erbα agonists that directly or indirectly target TGFβ signaling to mitigate the progression of PF [9,10,16].

Prior studies showed Rev-erbα agonists exhibited target-specific regulation of inflammatory response; for example, GSK4112 treatment suppressed LPS-induced IL-6 release in human myelomonocytic THP-1 cells *in vitro*, and SR9009 treatment reduced pro-inflammatory cytokine/chemokine IL-17A and IFN-γ in an experimental autoimmune encephalomyelitis mouse model *in vivo* [20,23]. Rev-erbα agonist GSK4112 treatment in primary human lung fibroblasts and IPF lung tissue explant cultures have been shown to reduce TGFβ1-induced activation of ECM genes *Acta2* and *Col1a1* [9]. In this study, we found that pre-treatment with different Rev-erbα agonists (GSK4112, SR9009, SR9011) before TGFβ1 treatment significantly reduced secretion of COL1A1 compared to TGFβ1 alone treated group. In contrast, pre-treatment with Rev-erbα antagonist SR8278 before TGFβ1 treatment augmented the secretion of COL1A1 in WI-38 cells.

Another report demonstrates that circadian clock controls secretory pathways and collagen homeostasis [24]. They showed novel clock modulators Rev-erbα agonist (SR9009) reduced collagen fibers/cell in wild-type (WT) and markedly reduced collagen fibers/cell in *Clock*^*∆19*^ fibroblasts and collagen accumulation. Additionally, Cry1/2 stabilizer (KL001) increased collagen fiber/cell in WT and decreased collagen fiber/cell in *Clock*^*∆19*^ fibroblasts [24]. These findings suggest that Rev-erbα is a critical factor that can regulate collagen synthesis and homeostasis in target cells, implicating Rev-erbα agonists as a novel small molecule that can regulate IPF pathobiology. Our findings corroborated with the earlier report [24] that Rev-erbα agonist (GSK4112) pre-treatment before TGFβ1 treatment significantly reduced intracellular FMT markers as confirmed by immunoblotting (GSK4112 reduced αSMA and COL1A1) and immunostaining followed by confocal microscopy (GSK4112, SR9009, and SR9011 reduced αSMA) in WI-38 cells. GSK4112 pre-treatment reduced TGFβ1-induced αSMA expression in primary human lung fibroblasts from healthy and IPF. Upregulation of αSMA is implicated in the progression of PF and our findings suggest that the Rev-erbα agonist reduced the αSMA production and suppressed the pro-fibrotic response. Nevertheless, Rev-erbα antagonist SR8278+TGFβ1 induces αSMA expression in WI-38 cells. Finally, we showed Rev-erbα agonist (GSK4112) pre-treatment markedly reduced TGFβ1-induced activation of pro-fibrotic genes (*Acta 2, Col1a1,* and *Fn-1),* but not Rev-erbα antagonist (SR8278). Lungs from IPF patients as well as the bleomycin-induced lung fibrosis in mice showed an increased pro-fibrotic mediator IL-6 implicated in the pathogenesis of PF [25,26]. We found Rev-erbα agonists (GSK4112) pre-treatment remarkably reduced TGFβ1-induced IL-6 release, while Rev-erbα antagonist (SR8278) pre-treatment instead augmented IL-6 release in WI-38 cells.

Overall, these promising findings were analogues to previous reports that had demonstrated the potential roles of Rev-erbα in regulating TGFβ-mediated canonical signaling during PF. Several different Rev-erbα agonists (GSK4112, SR9009 and SR9011) utilized in preclinical models were tested using human lung fibroblasts. GSK4112 showed markedly reduced pro-fibrotic phenotypes such as reduced protein abundance of αSMA and COL1A1, decreased transcript levels of fibrotic genes *Acta 2, Col1a1,* and *Fn-1,* and inhibits pro-inflammatory cytokine (IL-6) release in WI-38 cells. Rev-erbα antagonist SR8278 instead augmented TGFβ1-induced pro-fibrotic response. In conclusion, we showed that Rev-erbα agonists have great potential to reduce TGFβ1-mediated pro-fibrotic phenotypes. Inhibiting TGFβ signaling and collagen synthesis has been known to be one of the key mechanisms by which we can treat IPF pathobiology. These initial findings urge us to conduct further mechanistic studies using translationally relevant *in vitro*, *in vivo*, and *ex-vivo* models to determine the potential cell type-specific role of Rev-erbα in the progression of IPF.

## Supplementary Material

Supplemental figures
