# Supplementary material for: Rev-erbα agonists suppresses TGFβ1-induced fibroblast-to-myofibroblast transition and pro-fibrotic phenotype in human lung fibroblasts
Source: Biochem Biophys Res Commun. Author manuscript; Available in PMC 2024 Apr 22. (PMC11034855; doi:10.1016/j.bbrc.2023.05.092)
Supplement: Supplemental figures [file NIHMS1981328-supplement-Supplemental_figures.docx]

**Rev-erbα agonists suppresses TGFβ1-induced fibroblast-to-myofibroblast transition and pro-fibrotic phenotype in human lung fibroblasts**

Chandrashekhar Prasad^1^, Kameron Hahn^2^, Santosh Kumar Duraisamy^1^, Matthias A Salathe^1^, Steven K Huang^3^ Thomas P Burris^4^, and Isaac Kirubakaran Sundar^1,^*

^1^Department of Internal Medicine, Division of Pulmonary Critical Care and Sleep Medicine,

University of Kansas Medical Center, Kansas City, Kansas, USA.

^2^Division of Biological Sciences, University of Missouri, Columbia, MO, USA.

^3^ Department of Internal Medicine, Division of Pulmonary and Critical Care Medicine, University of Michigan, Ann Arbor, MI, USA

^4^College of Pharmacy, University of Florida, Gainesville, Florida, USA.


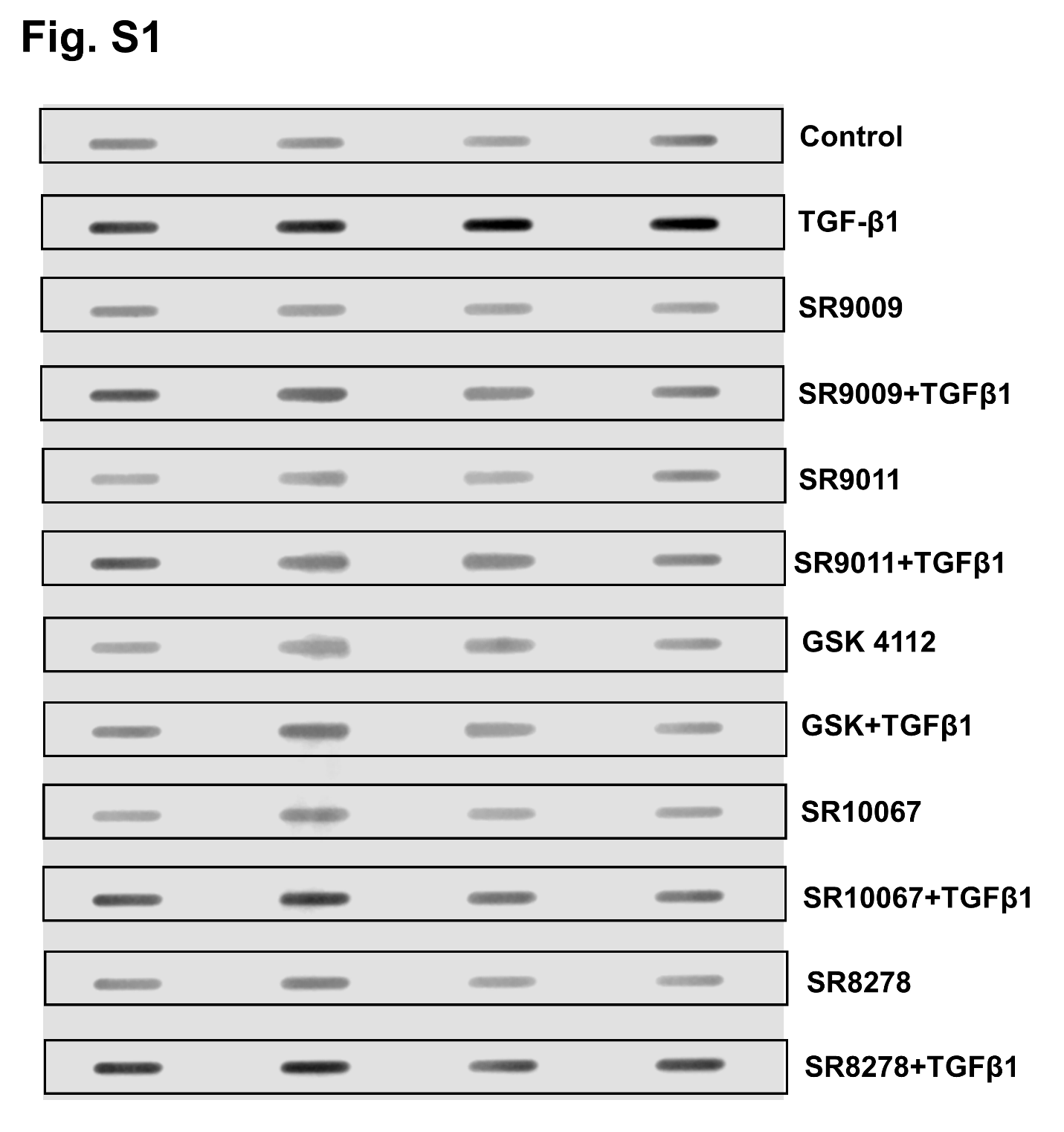


**Fig. S1. Rev-erbα agonist treatment inhibits TGFβ1-induced secretion of COL1A1 in WI-38 cells.**Uncropped/original Slot-Blot images showing secretory COL1A1 measured in conditioned medium of WI-38 cells pre-treated with and without Rev-erbα agonist (GSK4112/SR9009/SR9011; 10 µM) or antagonist (SR8278; 10 µM) for 4 hrs followed by TGFβ1 (10 ng/ml) stimulation for 48 hrs. We used the same control (untreated group) and TGFβ1 (treated group) for comparison with other Rev-erbα agonist/antagonist alone and Rev-erbα agonist/antagonist along with TGFβ1 treatment groups from the same experiment (see data presented in Fig. 1).


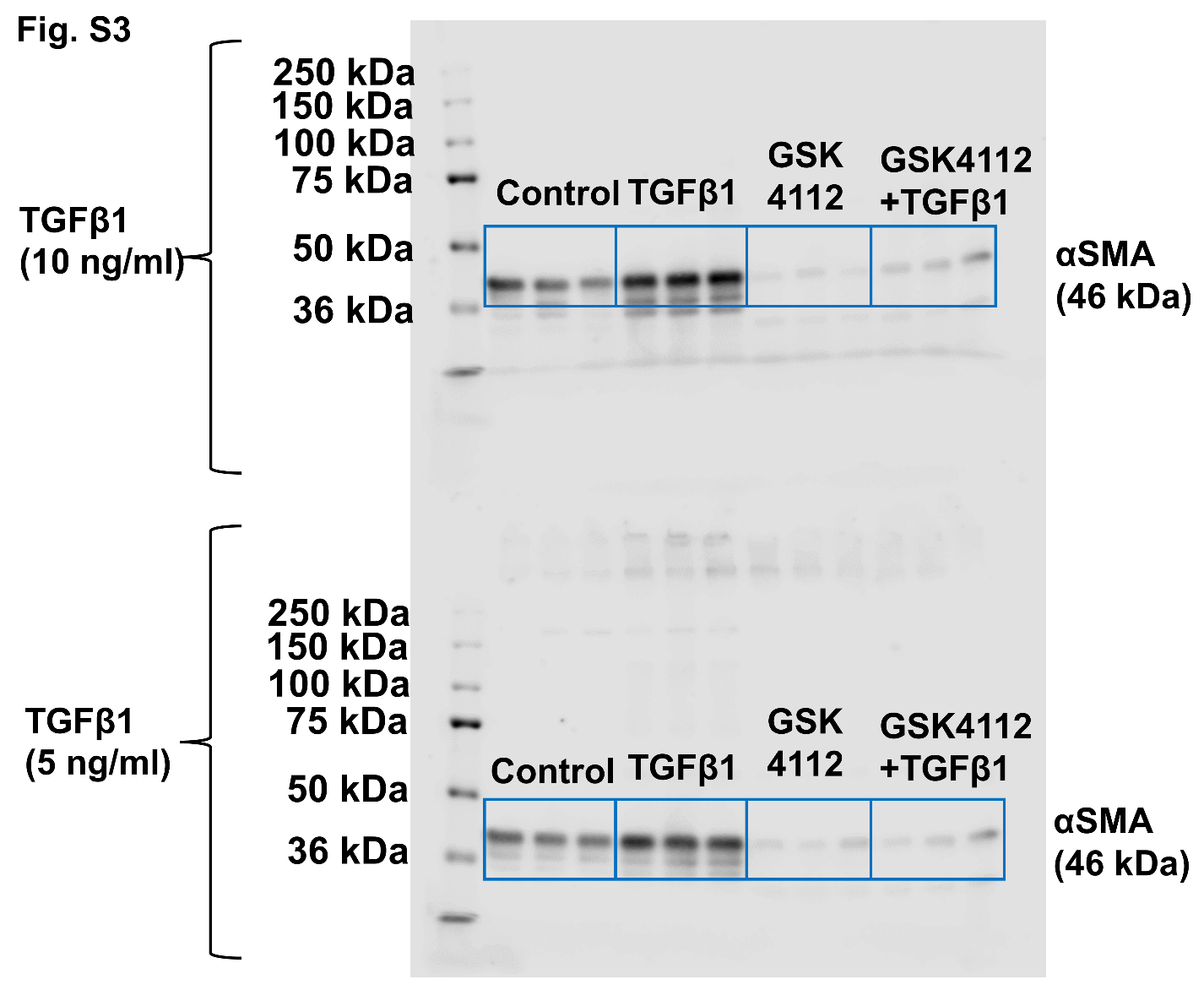


**Fig. S2. REV-ERBα** **agonist (GSK4112) inhibits TGFβ1-induced αSMA protein abundance in WI-38 cells.**Uncropped/original Western blot images showing αSMA protein abundance. Total protein isolated from WI-38 cells treated with TGFꞵ1 (10 ng/ml) or GSK4112 (20µM) or GSK4112 + 10 ng/ml TGFꞵ1 (Top blot); TGFꞵ1 (5 ng/ml) or GSK4112 (20µM) or GSK4112 + 5 ng/ml of TGFꞵ1 (Bottom blot) were separated on two separate 7.5% SDS-PAGE mini gels and transferred together on the same nitrocellulose membrane (top and bottom) and developed together.


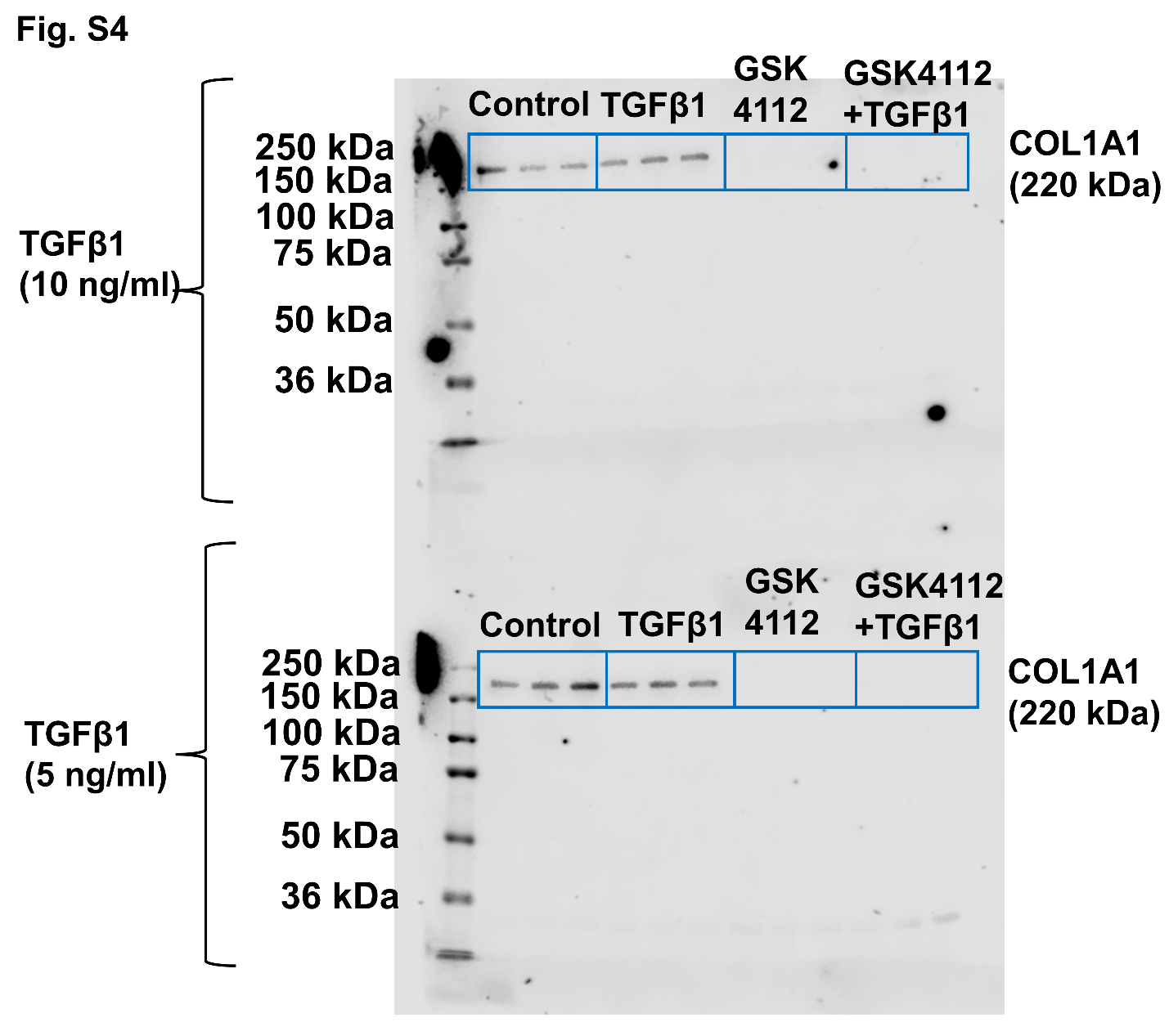


**Fig. S3. REV-ERBα** **agonist (GSK4112) inhibits TGFβ1-induced COL1A1 protein abundance in WI-38 cells.**Uncropped/original Western blot images showing COL1A1 protein abundance. Total protein isolated from WI-38 cells treated with TGFꞵ1 (10 ng/ml) or GSK4112 (20µM) or GSK4112 + 10 ng/ml TGFꞵ1 (Top blot); TGFꞵ1 (5 ng/ml) or GSK4112 (20µM) or GSK4112 + 5 ng/ml of TGFꞵ1 (Bottom blot) were separated on two separate 7.5% SDS-PAGE mini gels and transferred together on the same nitrocellulose membrane (top and bottom) and developed together.


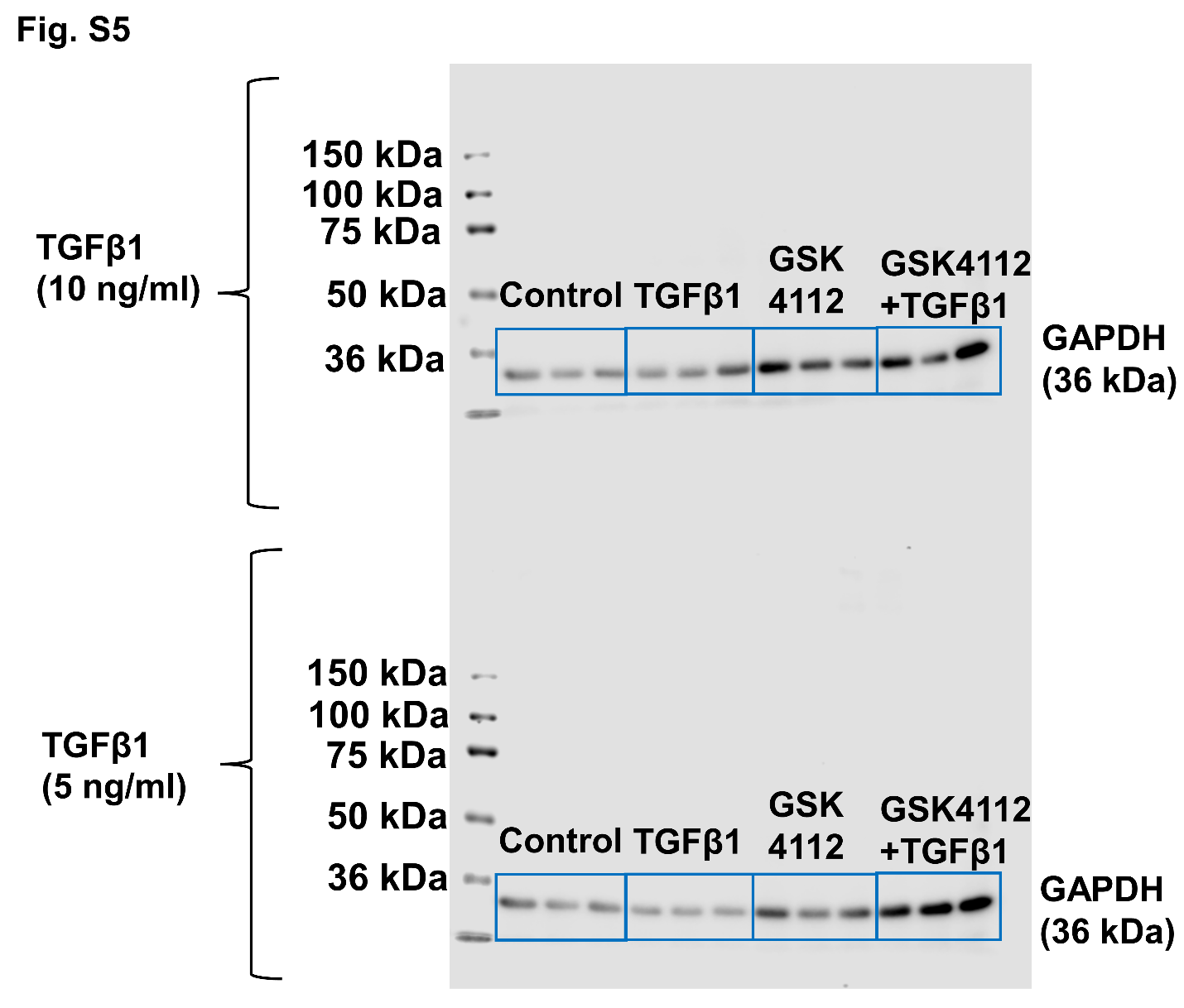


**Fig. S4. GAPDH housekeeping loading control for αSMA and COL1A1 protein abundance in WI-38 cells.**Uncropped/original Western blot images showing GAPDH protein abundance. Total protein isolated from WI-38 cells treated with TGFꞵ1 (10 ng/ml) or GSK4112 (20µM) or GSK4112 + 10 ng/ml TGFꞵ1 (Top blot); TGFꞵ1 (5 ng/ml) or GSK4112 (20µM) or GSK4112 + 5 ng/ml of TGFꞵ1 (Bottom blot) were separated on two separate 7.5% SDS-PAGE mini gels and transferred together on the same nitrocellulose membrane (top and bottom) and developed together.

| Human (Gene symbol) | Primer Sequence |
| --- | --- |
| *Col1a1* | F: 5'GAGAGCATGACCGATGGATT 3' |
|  | R: 5'CCTTCTTGAGGTTGCCAGTC 3' |
| *Acta2* | F: 5'CTGTTCCAGCCATCCTTCAT 3' |
|  | R: 5'CCGTGATCTCCTTCTGCATT 3' |
| *Fn-1* | F: 5'AATATCTCGGTGCCATTTGC 3' |
|  | R: 5'AAAGGCATGAAGCACTCAAT 3' |
| *Gapdh* | F: 5'CAATGACCCCTTCATTGACC 3' |
|  | R: 5'GACAAGCTTCCCGTTCTCAG 3' |

**Table S1. qRT-PCR primer sequences used in this study.**
